# Development of phytase-expressing chlamydomonas reinhardtii for monogastric animal nutrition

**DOI:** 10.1186/s12896-016-0258-9

**Published:** 2016-03-12

**Authors:** Fernanda Erpel, Franko Restovic, Patricio Arce-Johnson

**Affiliations:** Departamento de Genética Molecular y Microbiología, Pontificia Universidad Católica de Chile, Alameda 340, Santiago, Chile

**Keywords:** Microalgae, Phytase, Animal nutrition

## Abstract

**Background:**

In plant-derived animal feedstuffs, nearly 80 % of the total phosphorus content is stored as phytate. However, phytate is poorly digested by monogastric animals such as poultry, swine and fish, as they lack the hydrolytic enzyme phytase; hence it is regarded as a nutritionally inactive compound from a phosphate bioavailability point of view. In addition, it also chelates important dietary minerals and essential amino acids. Therefore, dietary supplementation with bioavailable phosphate and exogenous phytases are required to achieve optimal animal growth. In order to simplify the obtaining and application processes, we developed a phytase expressing cell-wall deficient *Chlamydomonas reinhardtii* strain.

**Results:**

In this work, we developed a transgenic microalgae expressing a fungal phytase to be used as a food supplement for monogastric animals. A codon optimized *Aspergillus niger* PhyA E228K phytase (*mE228K*) with improved performance at pH 3.5 was transformed into the plastid genome of *Chlamydomonas reinhardtii* in order to achieve optimal expression. We engineered a plastid-specific construction harboring the *mE228K* gene, which allowed us to obtain high expression level lines with measurable in vitro phytase activity. Both wild-type and cell-wall deficient strains were selected, as the latter is a suitable model for animal digestion. The enzymatic activity of the *mE228K* expressing lines were approximately 5 phytase units per gram of dry biomass at pH 3.5 and 37 °C, similar to physiological conditions and economically competitive for use in commercial activities.

**Conclusions:**

A reference basis for the future biotechnological application of microalgae is provided in this work. A cell-wall deficient transgenic microalgae with phytase activity at gastrointestinal pH and temperature and suitable for pellet formation was developed. Moreover, the associated microalgae biomass costs of this strain would be between US$5 and US$60 per ton of feedstuff, similar to the US$2 per ton of feedstuffs of commercially available phytases. Our data provide evidence of phytate-hydrolyzing microalgae biomass for use as a food additive without the need for protein purification.

## Background

Phytic acid (*myo*-inositol hexakisphosphate or IP6) is the major phosphate source in plant seeds, accounting for nearly 80 % of the total phosphate content. It is mostly found as phytate, and forms metal complexes with divalent cations, such as Ca^2+^, Mg^2+^, Zn^2+^ and Fe^2+^ [1, 2]. Monogastric animals are incapable of digesting phytate from plant-derived feeds, due to the lack of a phytase enzyme. Moreover, phytate exerts a strong chelating activity over positively-charged minerals and essential amino acids, reasons for which it is considered an important anti-nutrient [3, 4]. Therefore, in order to sustain animal nutritional needs, the livestock industry uses exogenous inorganic phosphate, greatly increasing total costs. Regarding this, during the last decade, the price of phosphate has risen sharply from US$200/t to US$1200/t, and is the third highest cost after energy and amino acids. From an ecological point of view, this agricultural practice is leading to an alarming increase in excreted phosphate levels and eutrophication of natural resources [5, 6]. Phytases (*myo*-inositol hexakisphosphate phosphohydrolases) on the other hand, are part of a phosphatase family that catalyzes the sequential dephosphorylation of phytate [7]. They are divided into two groups, according to phylogenetic analysis and biochemical properties: alkaline phytases, that have a strong preference for calcium-binding phytate; and the histidine acid phytases (HAP), which use mostly free phytate as substrate and show enzymatic activity at acid pH (between 2.5 and 6) and temperatures over 40 °C [8]. The application of phytases into the diet of monogastric animals has been adopted worldwide, not only with benefits for animal growth, but also for the environment. The use of HAPs has been directly correlated with a higher phosphate, mineral and essential amino acid bioavailability [3, 4]. Moreover, replacement of exogenous dicalcium phosphate by phytases, diminishes the phosphate concentration in swine excretions by 30–60 % [9]. An ideal phytase model should 1) be able to effectively hydrolyze phytate in order to obtain sufficient inorganic phosphate to supply the animal requirements, 2) be able to resist high temperatures in order to be incorporated into food pellets, and 3) have low production costs. Kim, Mullaney [10] developed single and multiple mutants of the *Aspergillus niger PHYA* gene, resulting in a single mutant (E228K) with a shifted optimum pH (from 5.5 to 3.8) and 266 % greater hydrolysis of phytate at pH 3.5 compared to the wild-type version, the same pH as found in the gastrointestinal system of monogastric animals.

On the other hand, microalgae have generated an enormous industrial interest as model systems for the production of added-value molecules. Among these, *Chlamydomonas reinhardtii* is the most recognized microalgae due to its suitability for genetic transformation and scalability; moreover, various examples of recombinant protein expression are available [11–14]. Improved recombinant protein production has been achieved by targeting via homologous recombination, specific plastid regions in order to obtain high gene expression, bypassing the known nuclear gene silencing that affects this organism [11, 12]. Several mutant strains of *C. reinhardtii* are available, among them the cell-wall deficient strain *cw15*, which is an excellent model for easily digestible microalgae [15]. Additionally, these organisms are an excellent source of carbohydrates, proteins and several nutrients, and have been accepted as **G**enerally **R**ecognized **A**s **S**afe (GRAS), ensuring their use in the food and medical industries [16]. From an economic standpoint, microalgae are low cost production bio-factories; estimated costs based on antibody production in mammal, plant and microalgae systems are US$150, US$0.005 and US$0.002, respectively [17]. Taken together, microalgae are thus excellent model organisms for use in animal feeds due to the ecological, economical and practical advantages. For these reasons, we expressed an optimized version of the *PHYA E228K* gene (hereafter named *mE228K*) in both wild-type and cell-wall deficient *cw15 C. reinhardtii* strains. Cell wall mutants could be useful in applications where cell disruption is needed, as for example, in animal nutrition. Phytase produced by microalgae effectively hydrolyzes phytate and should be able to resist high pelleting-like temperatures.

## Results & discussion

The potential of microalgae for producing high-value molecules, and their use in economically important activities as an oral administration vehicle have already been demonstrated [18]. *C. reinhardtii* has been extensively used as a model protein factory, and here we show how a cell-wall mutant (*cw15*) could be used in the administration of phytase into animal feedstuffs. We chose an improved version of the *PhyA* gene of *Aspergillus niger*, with a higher activity at pH 3.5, as found in the gastrointestinal system of monogastric animals [10].

### Codon optimization of the *PHYA E228K* gene

**Fig. 1:**
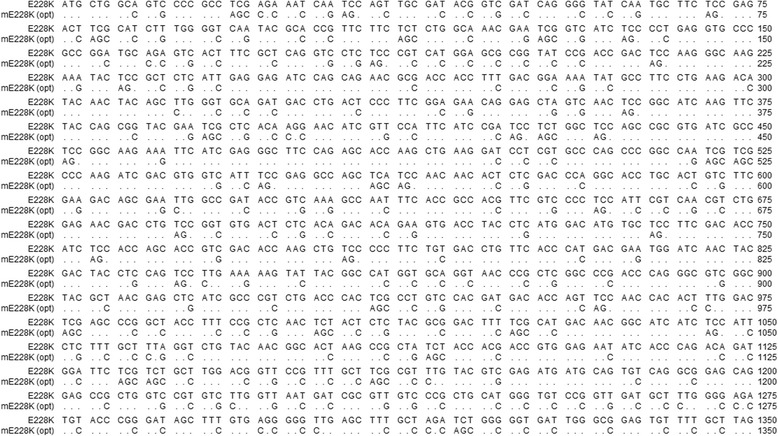
Codon optimization of the *E228K* gene; Codon optimization of the *E228K* gene described in Kim, Mullaney [10], based on the *Chlamydomonas reinhardtii* codon database [20]

We chose the PhyA E228K mutant, developed by Kim, Mullaney [10], as the phytase gene to express in *C. reinhardtii*. The wild-type version of PhyA has a double activity-peak pH profile, with two marked peaks at pH 2.5 and 5.5 [19]. However, the E228K mutant shifts the optimal pH to 3.8 and greatly increases phytate hydrolysis at pH 3.5, which is the typical pH of the digestive tract of monogastric animals [10]. In order to obtain an increased expression in *C. reinhardtii*, we performed codon optimization of the E228K mutant nucleotide sequence. A *C. reinhardtii* codon usage table was obtained from Nakamura, Gojobori [20], and this information was used in the OPTIMIZER webpage, where the optimization of the desired sequence was undertaken [21, 22]. It is worth noting that only the mature protein sequence was used, devoid of the extracellular destination N-terminal peptide, in order to trap the enzymatic activity within the intracellular space of the transformed microalgae. The resulting optimized gene (*mE228K*) was synthesized and cloned into a SmaI-digested pBluescript II SK(-) derivative lacking its multiple cloning sites (EPOCH Life Science, http://www.epochlifescience.com/). Further analysis of the optimized gene showed that more than half of the codons were optimized (51.56 %), whereas the sequences have a 78.1 % of nucleotide identity, and maintain 100 % amino acid identity. The detailed sequence information is shown in Fig. 1.

### Determination of transformation pressures

**Fig. 2:**
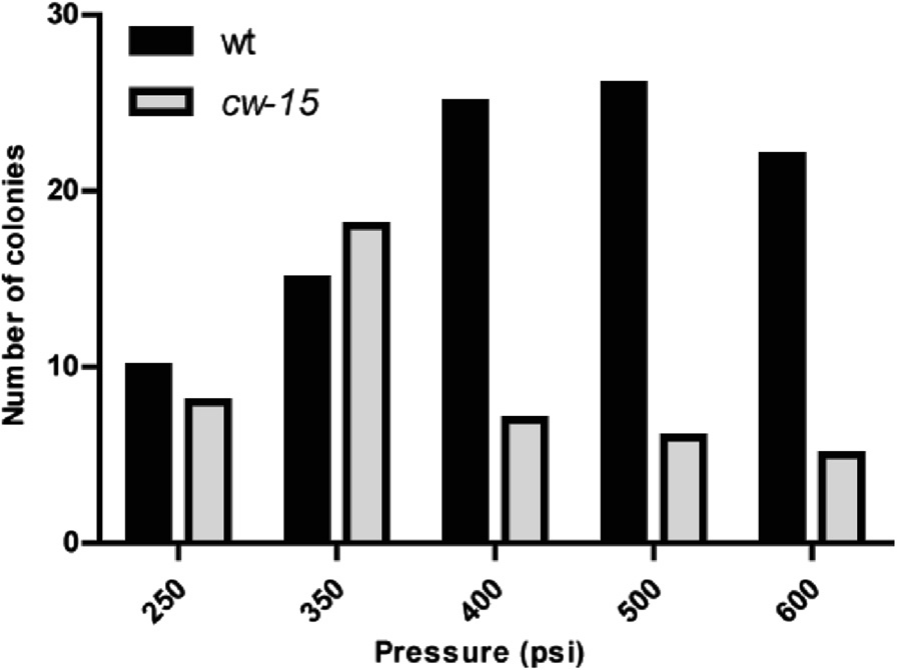
Optimization of biolistic transformation pressures for UTEX-90 and *cw15* strains; Total sum of kanamycin-resistant colonies from three transformation experiments for both UTEX-90 (*black bars*) and *cw15* (*grey bars*) strains, using pressures ranging from 250 to 600 psi

In order to optimize wild-type (UTEX-90) and *cw15* transformation, we subjected both strains to different shooting pressures of between 250 and 600 psi with an empty p3HB-Kan vector. Kanamycin resistant colonies were counted after incubation for 7 days in the presence of the antibiotic. As expected, more UTEX-90 colonies appeared at higher pressures (between 400 and 600 psi), whereas the cell-wall deficient strain was more efficiently-transformed at a lower pressure (near 350 psi; Fig. 2). Following this result, we determined 500 and 350 psi as the optimal shooting pressures for UTEX-90 and *cw15* strains, respectively.

### Cloning into the p3HB-Kan vector and transformation of *Chlamydomonas reinhardtii*

**Fig. 3:**
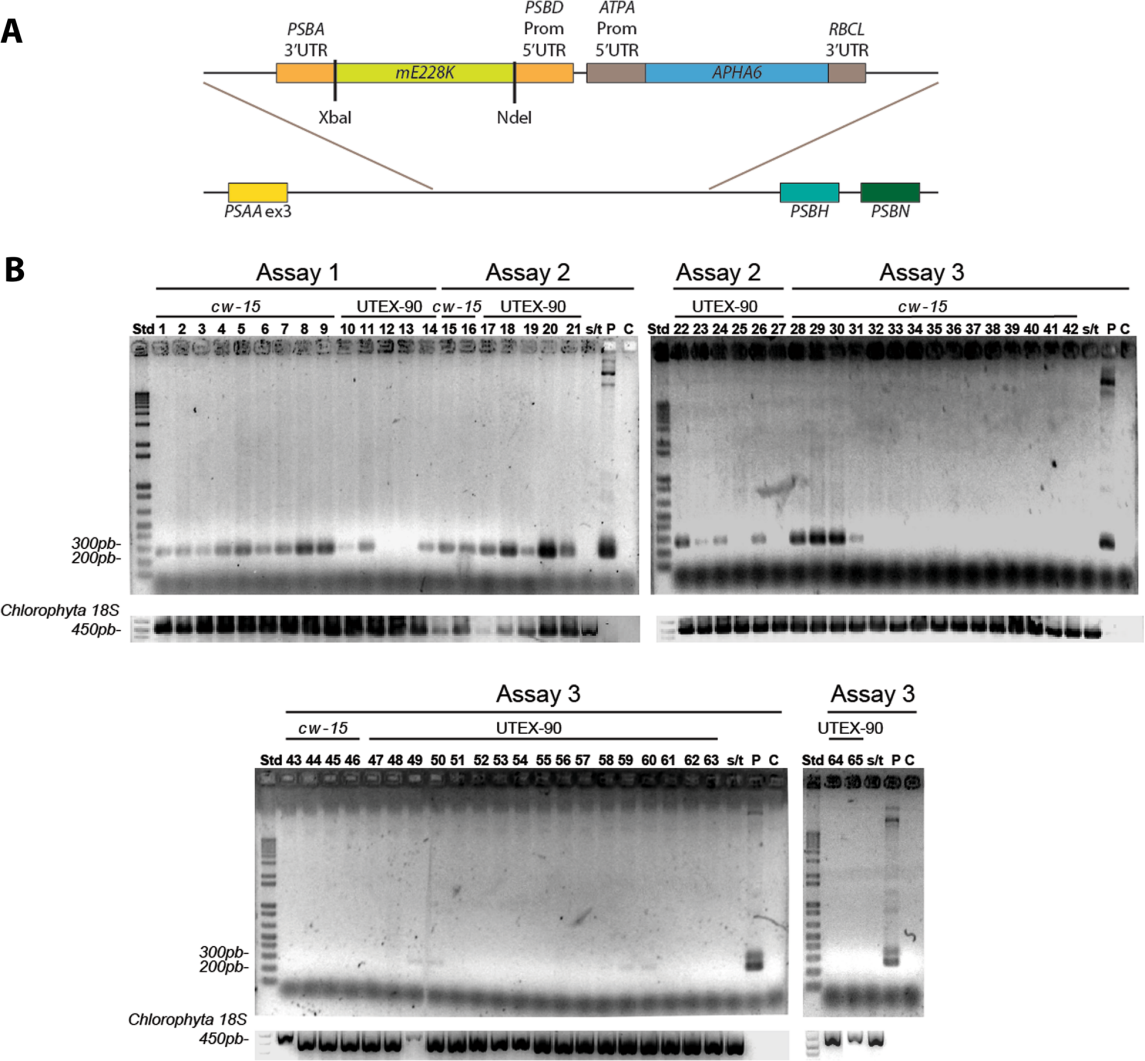
Transformation of *Chlamydomonas reinhardtii* with the *mE228K* gene; **a** Graphic representation of the homologous region of the p3HB-Kan-*mE228K* vector, which was inserted into the *Chlamydomonas reinhardtii* plastid genome in the *PSAA* ex3-*PSBH* intergenic regions. **b** Amplification of a vector region containing part of the *PSBD* promoter and the *mE228K* gene in kanamycin resistant colonies of transformed UTEX-90 (wild-type) and *cw15* strains. Three independent transformation assays are shown

The synthesized gene (*mE228K*) was cloned into the p3HB-Kan vector between the NdeI and XbaI sites [12]. The resulting vector (p3HB-Kan-*mE228K*) contains two regions of inverted repeats of the *C. reinhardtii* chloroplast genome (for homologous recombination) flanking a construction bearing the *PSBD* promoter/5′UTR, the optimized *mE228K* gene and the *PSBA* 3′UTR region, in addition to the *APHA6* gene flanked by the *ATPA* promoter/5′UTR and the *RBCL* 3′UTR, for kanamycin resistance (Fig. 3a). In order to transform *C. reinhardtii*, the p3HB-Kan-*mE228K* plasmid was coated onto nano-gold particles, and then delivered into cells of UTEX-90 and *cw15* strains plated in TAP-Agar plaques using a biolistic approach with shooting pressures of 500 and 350 psi, respectively. After two weeks of incubation at 16 h light/8 h dark and 24 °C in the presence of kanamycin, we obtained 65 resistant colonies, which were subsequently inoculated into 1 ml of liquid TAP medium without antibiotics for recovery. From these colonies, 30 were classified as stable transformants as determined by amplification of a vector region comprising part of the promoter and the *mE228K* gene, and at least two further sub-cultures in selective media (Fig. 3b).

### Expression of the *mE228K* transgene in transformed UTEX-90 and *cw15* strains

**Fig. 4:**
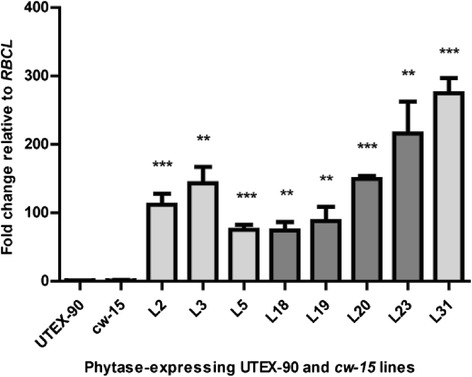
Expression levels of phytase-expressing UTEX-90 and *cw15* lines; RT-qPCR analysis of the *mE228K* transcript in transformed UTEX-90 (dark grey; control, L18, L19, L20 and L23) and *cw15* (light grey; control, L2, L3, L5 and L31) lines. The mRNA levels are shown as fold-change relative to the internal control *RBCL*. Asterisks indicate significant differences (*n* = 6 ± SEM; **: 0.01 > *p* > 0.001; ***: *p* < 0.001; all analysis compared to the non-transformed line)

To determine if kanamycin resistant colonies were expressing the *mE228K* transcript, we selected the four best-growing UTEX-90 (L18, L19, L20 and L23) and *cw15* (L2, L3, L5 and L31) transformed lines (data not shown) and analyzed them by RT-qPCR using *mE228K* specific primers. All transformed lines expressed the phytase gene between 75 and 275-fold compared to non-transformed controls (Fig. 4).

### Phytase activity determination

**Fig. 5:**
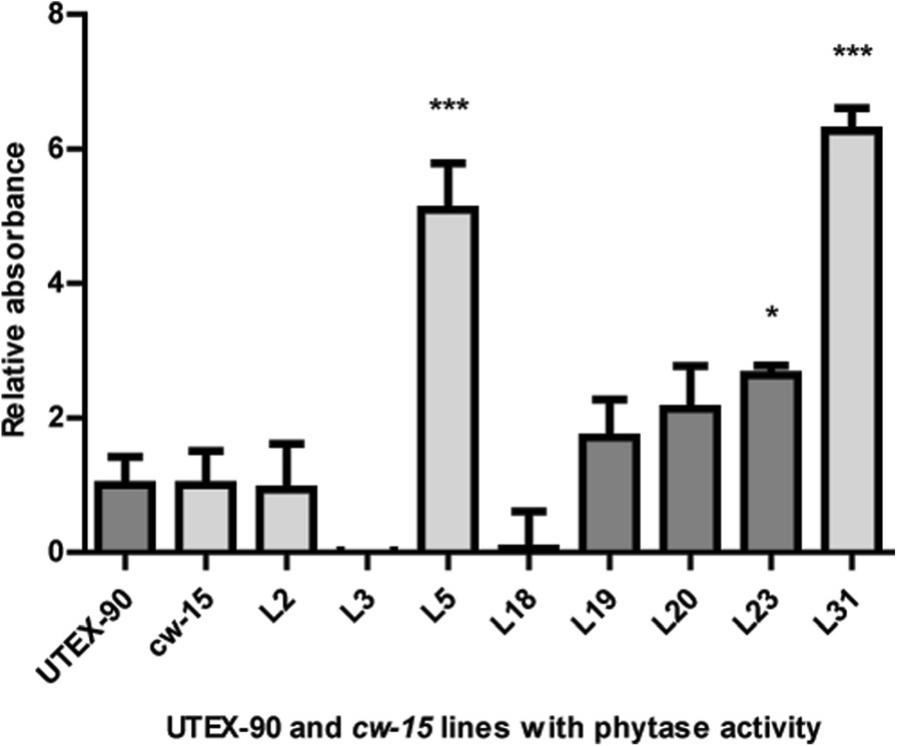
Determination of phytase activity of transformed UTEX-90 and *cw15* lines; Phytase activity determination of transformed lines of UTEX-90 (dark grey; control, L18, L19, L20 and L23) and *cw15* (light grey; control, L2, L3, L5 and L31) strains. The phytase levels are represented as fold-change relative to the mock control lacking lyophilized microalgae. Asterisks indicate significant differences (*n* = 6 ± SEM; *: 0.05 > *p* > 0.01; ***: *p* < 0.001; all analysis compared to the mock control)

A functional in vitro assay was performed in order to assess the enzymatic activity of the transformed microalgae lines. This method consisted in the detection of the released inorganic phosphate from exogenous phytic acid, which is measured colorimetrically. The total absorbance detected at 400 nm is directly proportional to the liberated inorganic phosphate. The microalgae lines with significant *mE228K* expression were lyophilized, and the resulting dry biomass was resuspended with phytate and assayed for phytase activity for 30 min at 37 °C at pH 3.5. We obtained three lines with significant phytase activity. Of these lines, two corresponded to *cw15* lines (L5 and L31) and the remaining to a UTEX-90 line (L23; Fig. 5).

Surprisingly, highest phytase activity (L5) did not correlate with higher gene expression, as transcript levels of L23 and L31 lines were approximately 2.8 and 3.6 fold higher than L5, respectively. Focusing on L5, the phytase activity of this line as determined at 37 °C and pH 3.5, was 5 units per gram of lyophilisate (μmols of free phosphate per 30 min * 0.1 mL of homogenate * 50 mg of lyophilisate dissolved in 1 mL of homogenate). Commercial product recommendations for phytase use in pig and chick feed are near 250–500 units per kg of feedstuffs (Natuphos®; taking into consideration that commercial units are determined at pH 4.5, and not 3.5, the actual digestive system pH of monogastric animals). This means that 50–100 g of lyophilized microalgae would be sufficient for every kg of feedstuffs (without considering the additional nutrients that microalgae biomass possess). Considering the costs of microalgae biomass production, estimates indicate that they would range between $50 and $600 per ton, with conservative yields of at least 20 g dry weight m^−2^ day^−1^ [18, 23]. Altogether, the associated microalgae biomass costs would be between US$5 and US$60 per ton of feedstuff, or even less at higher yields. These costs are relatively similar to the ones associated with phytase use in livestock feeding, which are near US$2 per ton of feedstuff [6]. Furthermore, *C. reinhardtii* (and microalgae in general) has high nutritional value, with lipid, carbohydrates and total protein contents close to 21, 17 and 48 % of its dry weight, respectively [24], meaning that the inclusion of microalgae biomass in feedstuffs could reduce the need to supplement feeds with other amino acids and necessary nutrients. In addition, *C. reinhardtii* is classified as GRAS, making this organism a suitable model for protein expression and commercialization [25, 26]. The utilization of the cell wall deficient strain *cw15* should additionally provide a more suitable substrate for animal digestion, which could mean a higher phytase bioavailability in the gastrointestinal system, without the need for protein purification.

## Conclusions

Phytase-expressing *C. reinhardtii* strains were developed to tackle the nutritional problems regarding phosphorus deficiency and general animal nutrition. The use of cell-wall deficient strains should become a viable option for delivering specific enzymatic activities in animal nutrition. Animal experiments are needed to confirm the in vivo phytase activity and to assess the safety and nutritional benefits associated with microalgae consumption.

## Methods

### Microalgae culture

The *Chlamydomonas reinhardtii* UTEX-90 (wild-type) and *cw15* strains were obtained from the Microalgae Culture Laboratory from the Universidad de Concepción and the Chlamydomonas Resource Center (http://www.chlamycollection.org/products/strains/), respectively. *C. reinhardtii* were grown in Tris-acetate-phosphate medium (TAP) at 25 °C, with a 16 h light/8 h dark photoperiod in an orbital shaker at 180 rpm with 1000 lux.

### Cloning and obtaining of transformed microalgae

The p3HB-Kan vector was a kind gift of Dr. Beth Rasala and Dr. Stephen Mayfield [11, 12]. Codon optimization of the *E228K* gene was performed using the *C. reinhardtii* codon database (http://www.kazusa.or.jp/codon/) described by Nakamura, Gojobori [20]. The resulting optimized *PHYA* gene (*mE228K*) was synthesized in the pBSK GS52811 vector containing an ampicillin resistance gene between the NdeI and XbaI restriction sites (Epoch Life Science). The *mE228K* gene was digested and subsequently cloned into p3HB-Kan, harboring the NdeI and XbaI sites. After cloning into p3HB-Kan, the insertion was confirmed by DNA sequencing.

Microalgae transformation by biolistics was performed as described in Rasala, Muto [11]. Briefly, UTEX-90 and *cw15* strains were cultivated in liquid TAP medium until the exponential growth stage (approximately 10^6^ cells/mL; DO_750_: 0.4). Later, cells were spun down, resuspended in TAP medium at a concentration of 10^9^ cells/mL, and 10 μL aliquots were cultivated in TAP-agar plates with 50 μg/mL kanamycin. Afterwards, plated cells were subjected to transformation using the Helios® Gene Gun (Bio-Rad, CA) with shooting pressures between 250 and 600 psi. The p3HB-Kan-*mE228K* vector was coated onto gold particles following manufacturer’s instructions. After transformation, cells were incubated for 7–10 days until growth of kanamycin-resistant colonies. The resistant colonies were inoculated into 1 mL of liquid TAP medium without antibiotic for recovery. Stable transformants were determined by Taq polymerase (Invitrogen) amplification of a vector region comprising part of the promoter and the *mE228K* gene (primers: 5′-TTGTTTTTTTATTTTGGAGATACACGCC-3′ and 5′-TCCTCGATCAGGGCGCTGTA-3′), and growth in at least two further sub-cultures in selective media.

### Transcript expression determination

Real time quantitative PCR (RT-qPCR) was performed in order to determine *mE228K* gene expression in transformed microalgae. Briefly, microalgae were homogenized in a Precellys® 24 (Berlin Technologies) and RNA was obtained using the TRIzol® reagent (Invitrogen). Then, 2 μg of RNA were treated with the Turbo DNA-Free^tm^ kit (Ambion®) and cDNA was made with random primers and Superscript II® Reverse Transcriptase (Invitrogen) following the manufacturer’s protocol. Quantification of the *mE228K* transcripts was performed using the Mx3000p^tm^ (Stratagene®) thermocycler and the SensiMix^tm^ Plus SYBR kit (Quantace) following manufacturer’s procedure. The *RBCL* gene was used as an expression normalizer, as described in Rasala, Muto [12]. Primers used were as follow: 5′-CCTGCAGAGCCTGAAGAAGT-3′ and 5′-CGAACAGGATGCTGATGATG-3′ for *mE228K*; 5′-CAGTTGCTTCAGGCGGTATT-3′ and 5′-AATTACGTCGCCACCTTCAC-3′ for *RBCL*.

### Phytase activity assay

Wild-type and phytase expressing cells were grown in 400 mL of selective media until the exponential growth phase (approximately 10^6^ cells). Cultures were centrifuged, washed with 1 % glycerol and centrifuged again. The resulting pellets were frozen with liquid nitrogen, thawed and frozen again previous to lyophilization in a Freezone 2.5 Benchtop Liter Lyophilizer (Labconco). The lyophilized whole-cell lysates (dry microalgae biomass) were used for phytase activity assays.

Phytase activity quantification was performed as described by Heinonen and Lahti [27] with modifications. Briefly, 25 mg of lyophilized microalgae were dissolved in 500 μL of 200 mM pH 3.5 sodium acetate – acetic acid buffer. In order to start the catalytic reaction, 100 μL of each sample were added to 500 μL of a 44.1 mM sodium phytate solution (in acetate buffer) previously incubated at 37 °C for 5 min. The mock was performed under the same conditions without the lyophilized microalgae, and a positive control was carried out adding 100 μL of a commercial phytase solution (Natuphos®). After incubation for 30 min at 37 °C, 4 mL of CRS colorimetric solution (acetone, 5 N sulphuric acid and 5 % ammonium molybdate; 2:1:1 v/v/v) was added, and absorbance (400 nm) was measured. A standard curve of absorbance was made with phosphate (0.5–2.5 μmol). The enzymatic activity (units per mg of lyophilisate) was obtained as the μmols of released phosphate per duration of assay, volume and lyophilisate mass.

## Availability of data and materials

The dataset supporting the conclusions of this article is available in the GenBank (National Center for Biotechnology Information) repository KT899873.

## References

[1] Reddy NR, Sathe SK, Salunkhe DK (1982). Phytates in legumes and cereals. Adv Food Res.

[2] Urbano G, Lopez-Jurado M, Aranda P, Vidal-Valverde C, Tenorio E, Porres J (2000). The role of phytic acid in legumes: antinutrient or beneficial function?. J Physiol Biochem.

[3] Rao DE, Rao KV, Reddy TP, Reddy VD (2009). Molecular characterization, physicochemical properties, known and potential applications of phytases: An overview. Crit Rev Biotechnol.

[4] Kumar V, Sinha A, Makkar H, Becker K (2010). Dietary roles of phytate and phytase in human nutrition: a review. Food Chem.

[5] Vats P, Banerjee UC (2005). Biochemical characterisation of extracellular phytase (myo-inositol hexakisphosphate phosphohydrolase) from a hyper-producing strain of Aspergillus niger van Teighem. J Ind Microbiol Biotechnol.

[6] Lei X, Weaver J, Mullaney E, Ullah A, Azain M (2013). Phytase, a new life for an “Old” enzyme. Annu Rev Anim Biosci.

[7] Wyss M, Brugger R, Kronenberger A, Remy R, Fimbel R, Oesterhelt G (1999). Biochemical characterization of fungal phytases (myo-inositol hexakisphosphate phosphohydrolases): catalytic properties. Appl Environ Microbiol.

[8] Oh BC, Choi WC, Park S, Kim YO, Oh TK (2004). Biochemical properties and substrate specificities of alkaline and histidine acid phytases. Appl Microbiol Biotechnol.

[9] NRC (1998). Nutrient Requirements of Swine: 10th Revised Edition.

[10] Kim T, Mullaney EJ, Porres JM, Roneker KR, Crowe S, Rice S (2006). Shifting the pH profile of Aspergillus niger PhyA phytase to match the stomach pH enhances its effectiveness as an animal feed additive. Appl Environ Microbiol.

[11] Rasala BA, Muto M, Lee PA, Jager M, Cardoso RM, Behnke CA (2010). Production of therapeutic proteins in algae, analysis of expression of seven human proteins in the chloroplast of Chlamydomonas reinhardtii. Plant Biotechnol J.

[12] Rasala BA, Muto M, Sullivan J, Mayfield SP (2011). Improved heterologous protein expression in the chloroplast of Chlamydomonas reinhardtii through promoter and 5′ untranslated region optimization. Plant Biotechnol J.

[13] Tran M, Zhou B, Pettersson PL, Gonzalez MJ, Mayfield SP (2009). Synthesis and assembly of a full-length human monoclonal antibody in algal chloroplasts. Biotechnol Bioeng.

[14] Wang X, Brandsma M, Tremblay R, Maxwell D, Jevnikar AM, Huner N (2008). A novel expression platform for the production of diabetes-associated autoantigen human glutamic acid decarboxylase (hGAD65). BMC Biotechnol.

[15] Wang ZT, Ullrich N, Joo S, Waffenschmidt S, Goodenough U (2009). Algal lipid bodies: stress induction, purification, and biochemical characterization in wild-type and starchless Chlamydomonas reinhardtii. Eukaryot Cell.

[16] Gantar M, Svircev Z (2008). Microalgae and cyanobacteria: food for thought. J Phycol.

[17] Mayfield SP, Franklin SE, Lerner RA (2003). Expression and assembly of a fully active antibody in algae. Proc Natl Acad Sci U S A.

[18] Specht E, Miyake-Stoner S, Mayfield S (2010). Micro-algae come of age as a platform for recombinant protein production. Biotechnol Lett.

[19] Ullah AH, Gibson DM (1987). Extracellular phytase (E.C. 3.1.3.8) from Aspergillus ficuum NRRL 3135: purification and characterization. Prep Biochem.

[20] Nakamura Y, Gojobori T, Ikemura T (2000). Codon usage tabulated from international DNA sequence databases: status for the year 2000. Nucleic Acids Res.

[21] Puigbo P, Romeu A, Garcia-Vallve S (2008). HEG-DB: a database of predicted highly expressed genes in prokaryotic complete genomes under translational selection. Nucleic Acids Res.

[22] Puigbo P, Guzman E, Romeu A, Garcia-Vallve S (2007). OPTIMIZER: a web server for optimizing the codon usage of DNA sequences. Nucleic Acids Res.

[23] Williams PJB, Laurens LML (2010). Microalgae as biodiesel & biomass feedstocks: review & analysis of the biochemistry, energetics & economics. Energy Environ Sci.

[24] Becker EW (2007). Micro-algae as a source of protein. Biotechnol Adv.

[25] Mayfield SP, Franklin SE (2005). Expression of human antibodies in eukaryotic micro-algae. Vaccine.

[26] Mayfield SP, Manuell AL, Chen S, Wu J, Tran M, Siefker D (2007). Chlamydomonas reinhardtii chloroplasts as protein factories. Curr Opin Biotechnol.

[27] Heinonen JK, Lahti RJ (1981). A new and convenient colorimetric determination of inorganic orthophosphate and its application to the assay of inorganic pyrophosphatase. Anal Biochem.

